# Relapsed Multiple Myeloma in the Gastrointestinal Tract With Aberrant Expression of CD3: A Case Report

**DOI:** 10.7759/cureus.75336

**Published:** 2024-12-08

**Authors:** Priscilla Quach, Michael Lack, Ryan M Ash, Mark T Cunningham, Daniel Farrell

**Affiliations:** 1 Pathology and Laboratory Medicine, University of Kansas Medical Center, Kansas City, USA; 2 Radiology, University of Kansas Medical Center, Kansas City, USA

**Keywords:** extramedullary multiple myeloma, gastrointestinal multiple myeloma, multiple myeloma with aberrant cd3, plasma cell myeloma, relapsed multiple myeloma

## Abstract

This report describes a rare case of relapsed multiple myeloma in the gastrointestinal tract with aberrant CD3 expression. Upon admission for acute renal failure, the patient had abnormal computed tomography scan findings of the abdomen and pelvis. Subsequent colonoscopy found numerous polyps and masses. Histologic examination of a biopsy of one of the masses revealed a diffuse atypical cellular infiltrate with undifferentiated morphology in the submucosa that was positive for CD3, CD4, weak and variable CD79a, CD138, BCL2, CMYC, MUM1, vimentin, and monoclonal cytoplasmic lambda. A diagnosis of recurrent plasma cell myeloma was made. The positive expression of T-cell markers CD3 and CD4 may cause confusion with T-cell lymphoma in a scenario with undifferentiated morphology as in our case.

## Introduction

Multiple myeloma (MM) is a hematologic neoplasm predominantly affecting the elderly population (median age of 70 years old), comprising approximately 10% of all hematologic malignancies [1], and is characterized by clonal expansion of plasma cells. The disease course is typically marked by multiple relapses and remissions with each subsequent remission having a shorter duration. MM classically presents with hypercalcemia, renal damage, anemia, and bone lesions (CRAB signs and symptoms). In 2014, the International Myeloma Working Group (IMWG) updated diagnostic criteria to include greater than or equal to sixty percent clonal bone marrow plasma cells, serum free light chain (FLC) ratio of 100 or more with the involved FLC being 100 mg/L or higher, and more than one focal bone lesion present on magnetic resonance imaging (MRI) (SLiM criteria) [2].

However, in the absence of systemic signs of bone marrow involvement, patients may also present with plasmacytomas: local collections of neoplastic plasma cells present either in bone or an extramedullary site. Most solitary plasmacytomas of bone occur in the axial skeleton, with the spine most commonly involved. The most common location for extramedullary plasmacytomas is the upper respiratory tract; other less common locations include lymph nodes, lungs, gastrointestinal (GI) tract, genitourinary tract, and skin [3].

Typical histologic features of plasma cells include an eccentrically located nucleus with mature, clumped chromatin in a “clockface” pattern and abundant cytoplasm. However, in cases with undifferentiated or plasmablastic morphology, plasmacytoid features may be sparse. Plasma cells are of B-cell lineage and, immunophenotypically, normally express the B-cell lineage defining marker CD19 while being negative for CD20. In contrast, neoplastic plasma cells as seen in MM are usually aberrantly negative for CD19 and/or aberrantly positive for CD20, CD56, or CD117 and monoclonal cytoplasmic kappa or lambda light chain. Both benign and neoplastic plasma cells express typical plasma cell markers such as CD38 and CD138 [4].

CD3, a marker commonly thought of as T-cell lineage defining, is rarely reported as being aberrantly expressed by mature B-cell neoplasms, including MM. Infrequent CD3 expression in these neoplasms is most likely due to the absence of T-cell genetic programming in normal mature B cells and normal plasma cells. This lineage infidelity by CD3 can pose a diagnostic challenge. Pathologists must be vigilant in distinguishing aberrant CD3 expression in B-cell neoplasms from true T-cell lymphomas, particularly when faced with undifferentiated morphology or when the patient's history is unavailable.

## Case presentation

**Figure 1:**
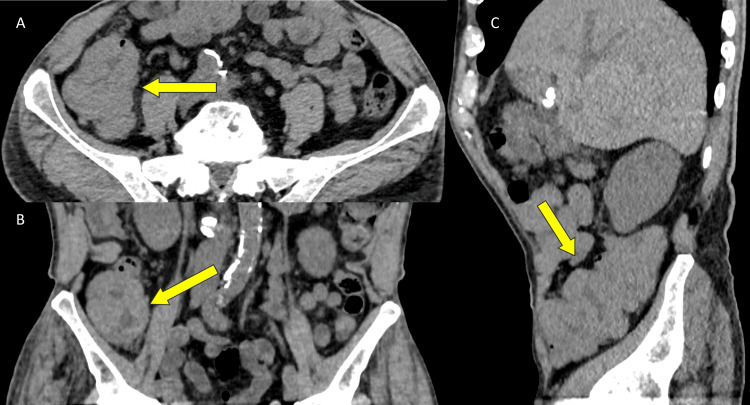
A. Axial, B. Coronal, and C. Sagittal images from a non-contrast CT exam demonstrating mural wall thickening of the ascending colon (yellow arrows)

**Figure 2:**
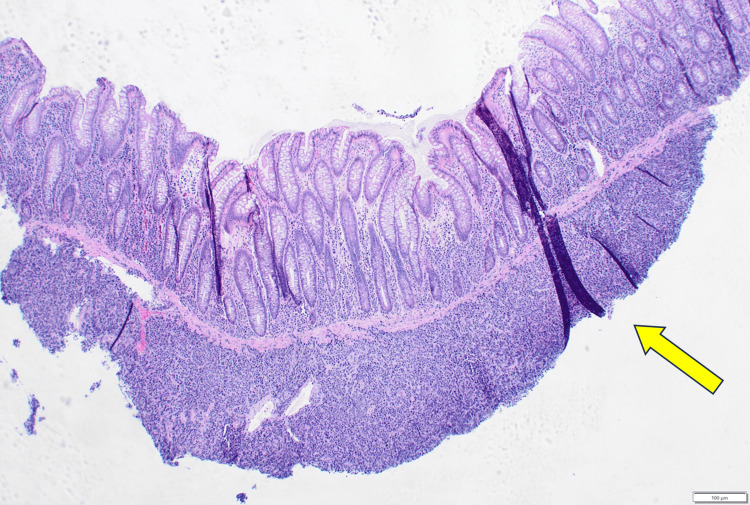
Colon polyp biopsy with atypical cellular infiltrate in submucosa (yellow arrow, Hematoxylin and eosin stain, 40x)

**Figure 3:**
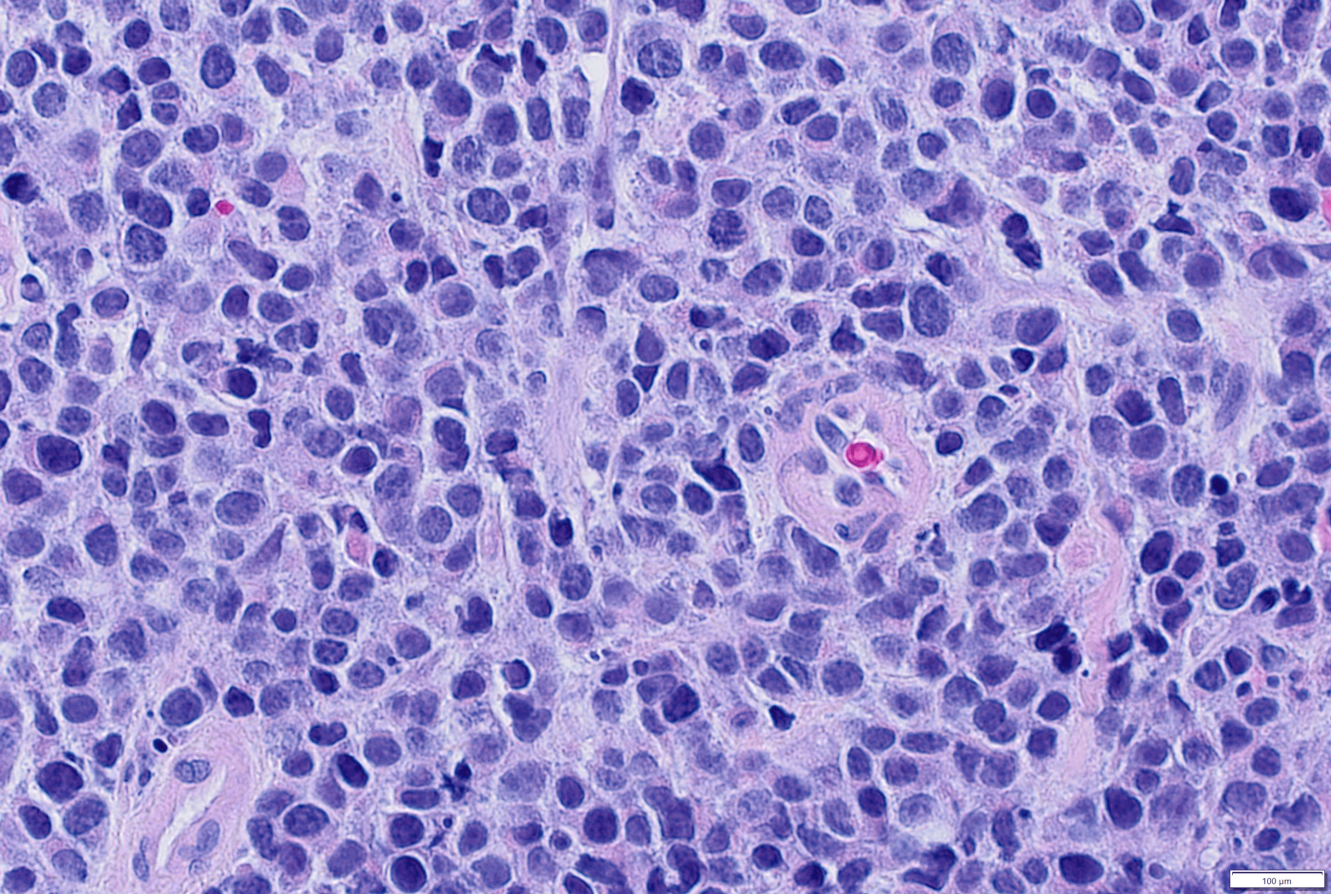
Colon polyp biopsy with atypical cellular infiltrate (hematoxylin and eosin stain, 500x oil)

An 80-year-old male patient was admitted to the Hematology Clinic for anemia requiring transfusion and acute renal failure after his creatinine was discovered to be elevated at 5.86 mg/dL (reference range 0.4-1.24 mg/dL) with an estimated creatinine clearance less than 10 ml/min. His past medical history was significant for MM (diagnosed 13 years prior) status-post stem cell transplant and maintenance therapy, as well as B-lymphoblastic leukemia/lymphoma (diagnosed three years prior) status-post treatment (completed one year prior) and in remission. The physical exam was unremarkable. Laboratory studies are summarized in Table 1. A CT scan of the abdomen/pelvis showed an irregular thickening of the cecum and ascending colon, concerning for primary colorectal malignancy versus extraosseous plasmacytoma (Figure 1). Colonoscopy demonstrated multiple polyps and masses up to 4 cm in size throughout the entire colon, some with ulcerations but without bleeding, most concentrated in the cecum and ascending colon. Multiple biopsies of the polyps and masses were taken and sent for histologic examination. Low-power light microscopy examination showed a diffuse atypical cellular infiltrate within the colonic submucosa (Figure 2). High-power light microscopy examination showed that the atypical cells had an overall undifferentiated morphology with medium-to-large nuclei, dispersed chromatin, occasional prominent nucleoli, and variable amounts of cytoplasm (Figure 3).

**Table 1:** Pertinent laboratory results

Laboratory Study	Result	Reference Range
Hemoglobin	6.9 g/dL	13.5-16.5 g/dL
Platelets	128 k/uL	150-400 k/uL
White blood cells	5.3 k/uL	4.5-11.0 k/uL
Sodium	134 mmol/L	137-147 mmol/L
Potassium	5.2 mmol/L	3.5-5.1 mmol/L
Chloride	103 mmol/L	98-110 mmol/L
CO2	18 mmol/L	21-30 mmol/L
Anion gap	13	3-12
Blood urea nitrogen	73 mg/dL	7-25 mg/dL
Creatinine	5.86 mg/dL	0.4-1.24 mg/dL
eGFR	9 mL/min	> 60 mL/min
Glucose	102 mg/dL	70-100 mg/dL
Albumin	3.0 g/dL	3.5-5.0 g/dL
Calcium	8.9 mg/dL	8.5-10.6 mg/dL

**Figure 4:**
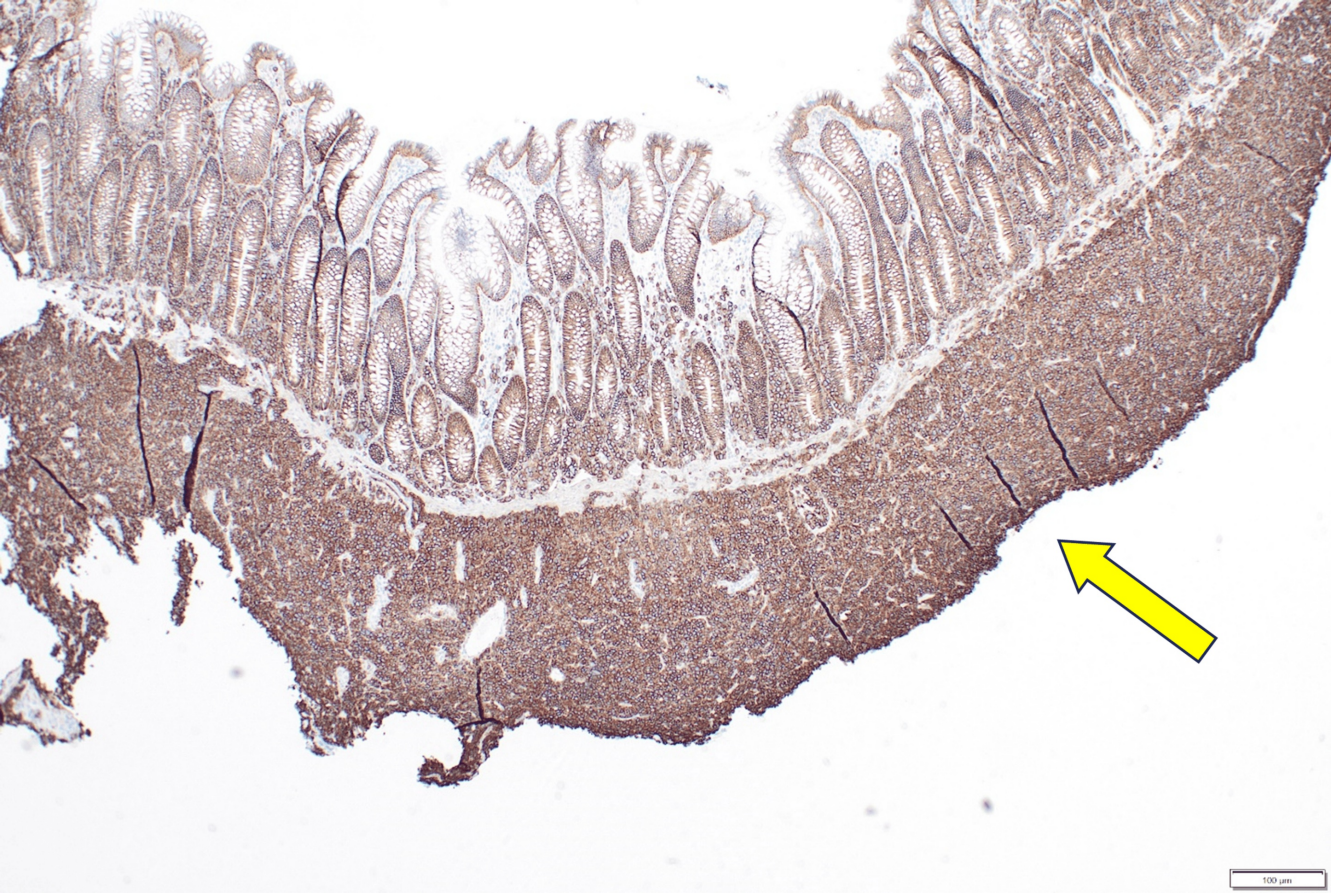
Immunohistochemistry for CD138, diffuse positivity in atypical cells (40x)

**Figure 5:**
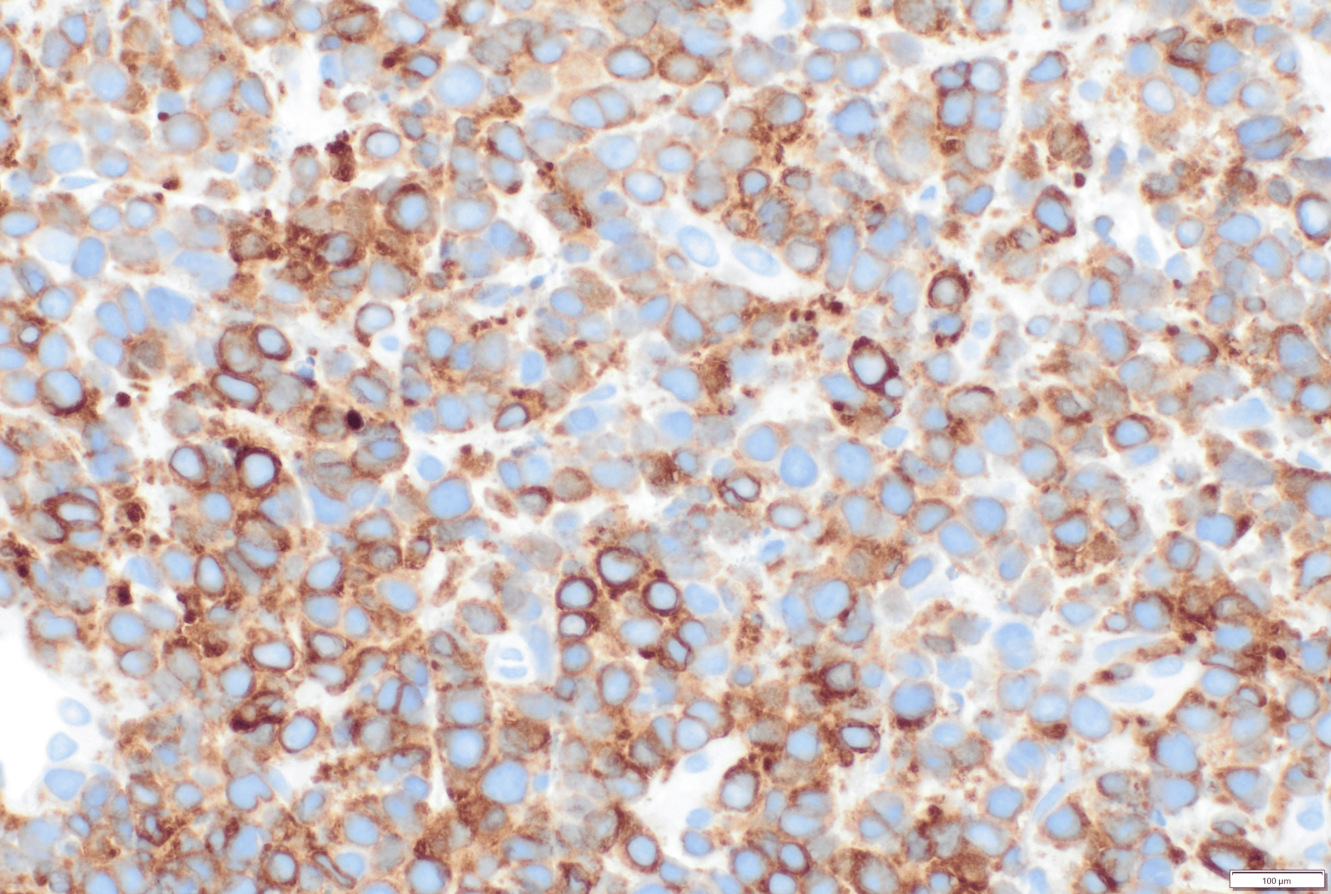
Immunohistochemistry for CD3, positivity in atypical cells (500x oil)

**Figure 6:**
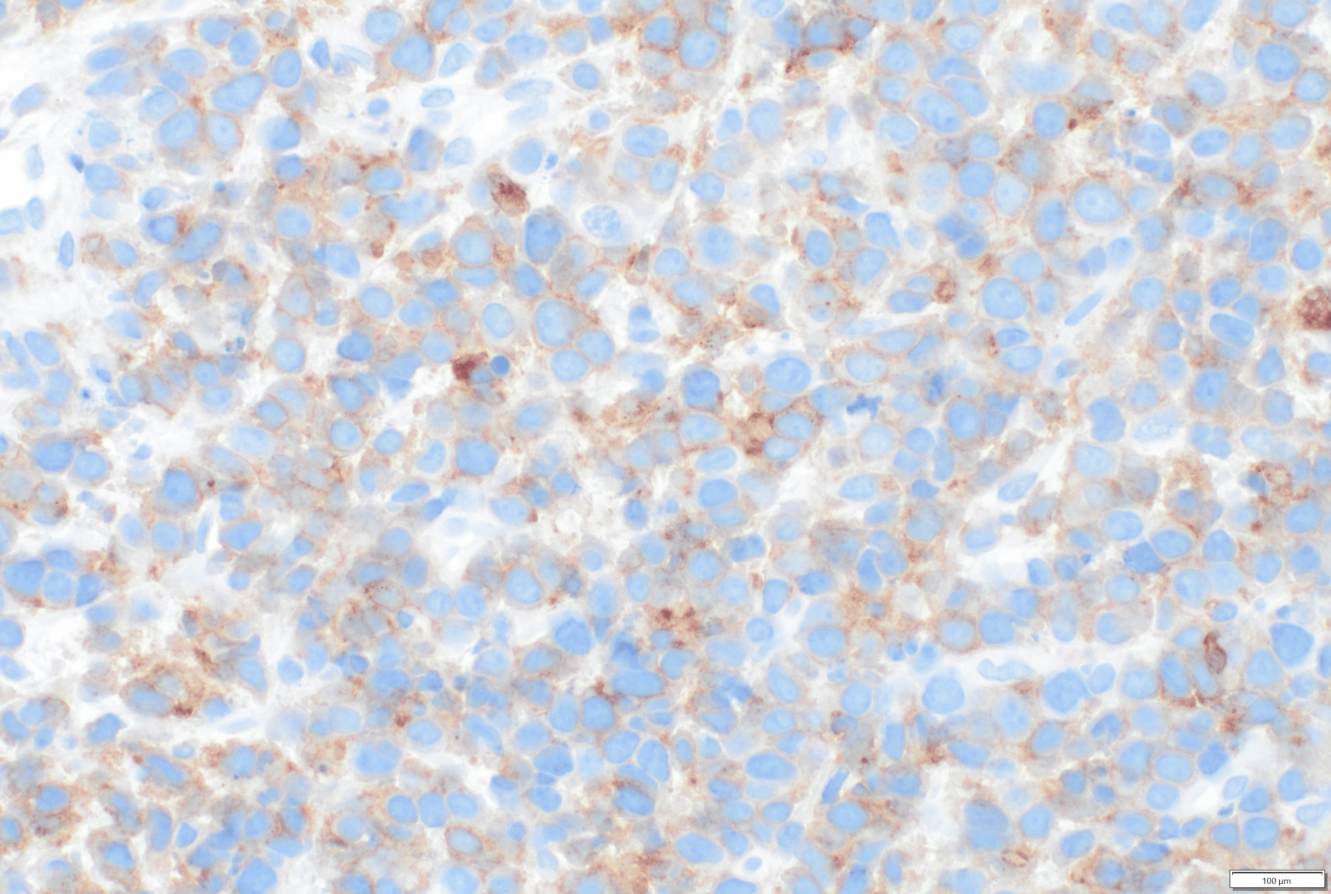
Immunohistochemistry for CD4, variable positivity in atypical cells (500x oil)

**Figure 7:**
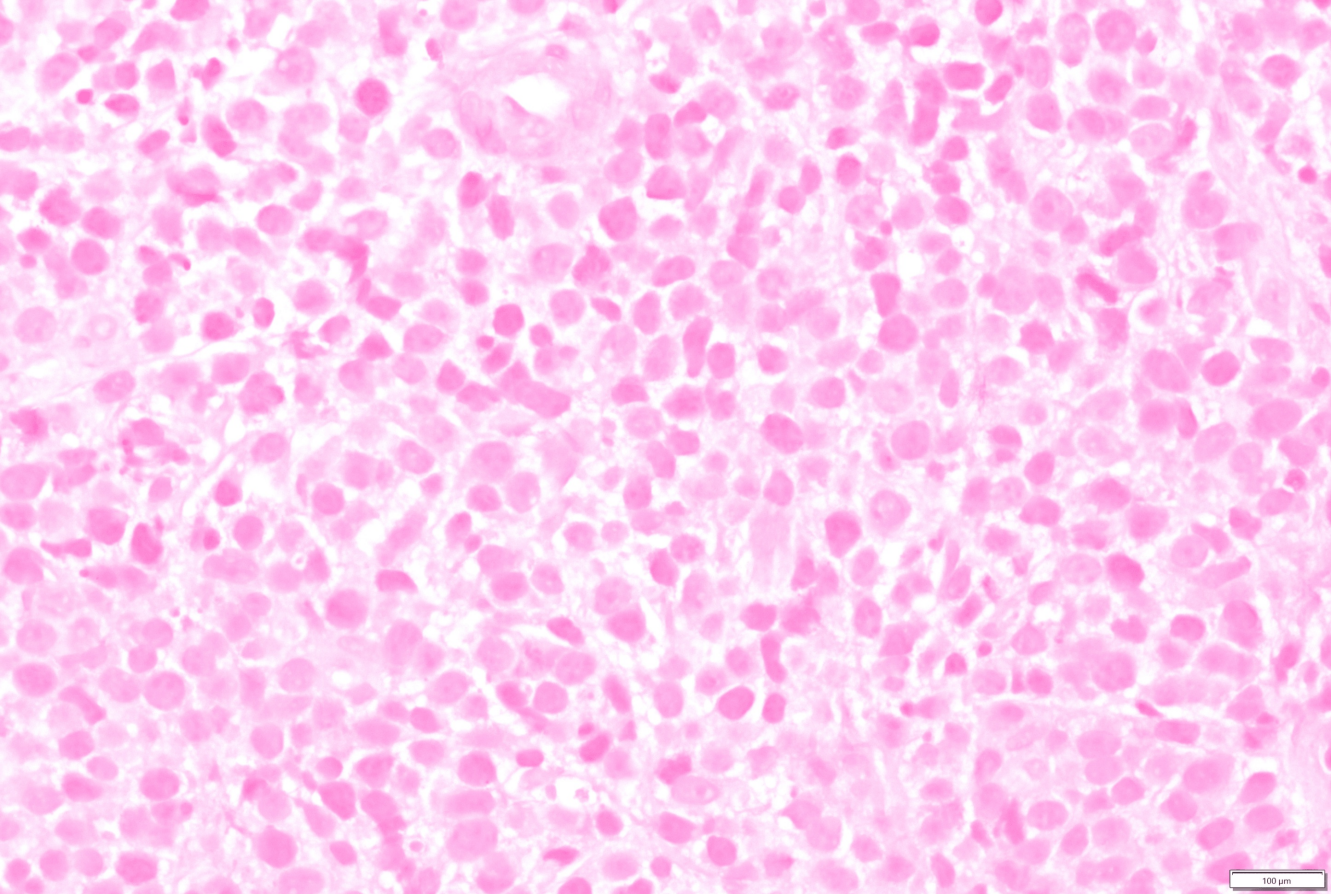
Chromogenic in situ hybridization for kappa light chain, negative in atypical cells (500x oil)

**Figure 8:**
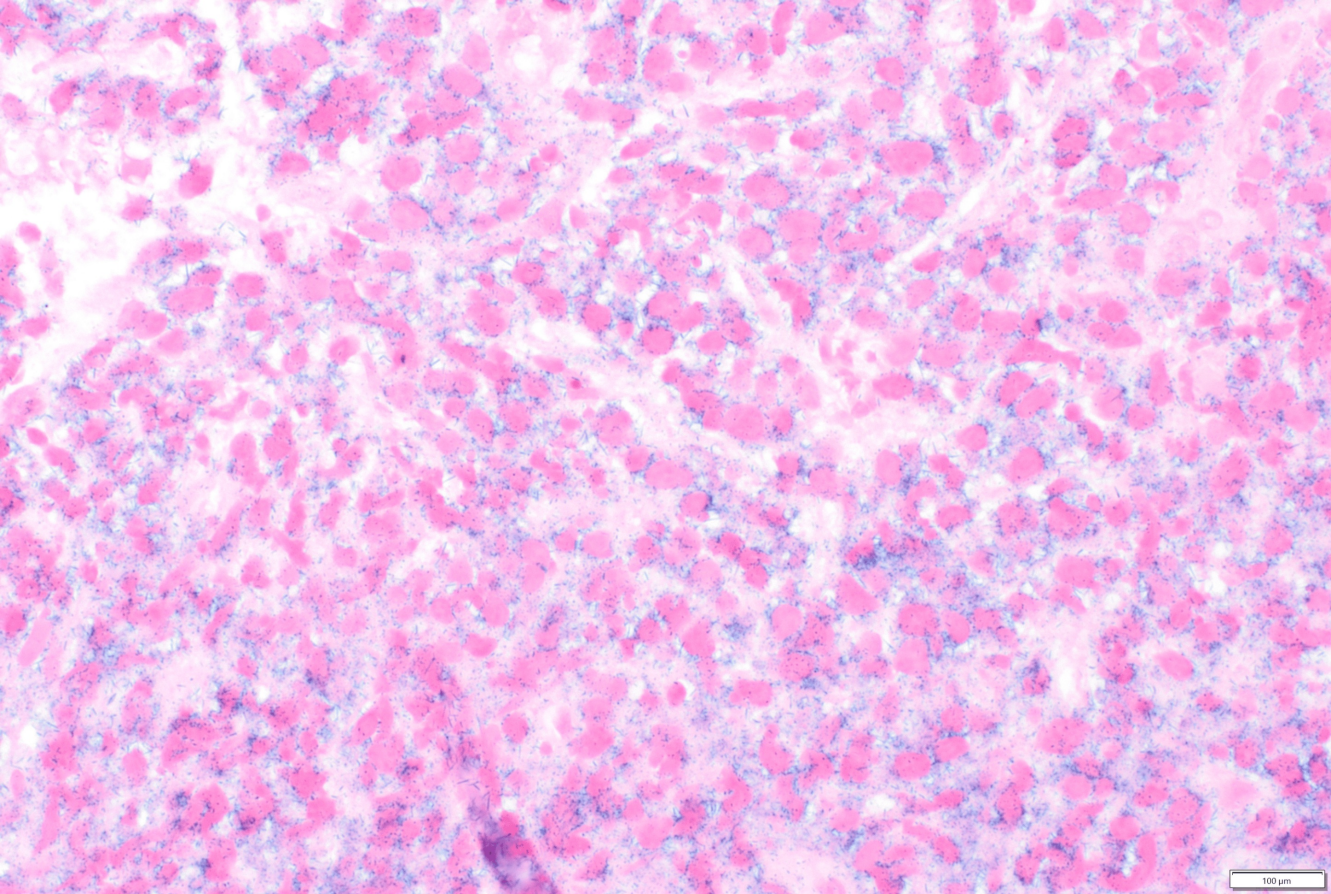
Chromogenic in situ hybridization for lambda light chain, positivity in atypical cells (500x oil)

Immunohistochemical stains were performed for the following markers: CD1a, CD2, CD3, CD4, CD5, CD7, CD8, CD10, CD19, CD20, CD30, CD33, CD34, CD45, CD56, CD79a, CD117, CD138, ALK1, BCL2, BCL6, CAM5.2, CMYC, cyclin D1, granzyme B, Ki67, MUM1, myeloperoxidase, pancytokeratin, TdT, TIA1, and vimentin. The atypical cells were positive for CD3 (diffuse, cytoplasmic), CD4, CD79a (variable, up to 10% multifocally), CD138 (strong, diffuse), BCL2, CMYC, MUM1, and vimentin. The Ki-67 proliferation index was markedly elevated at 95%. The remainder of the markers were negative in the atypical cells. Chromogenic in situ hybridization stains for kappa and lambda light chain were performed, and the atypical cells were found to have monotypic lambda light chain expression. Stains for CD3 and CD4 were repeated on the remainder of the tissue blocks and confirmed that the atypical cells were positive for both CD3 and CD4 (variable). Chromogenic in situ hybridization for Epstein-Barr virus (EBV) was negative. Pertinent stains are shown in Figures 4-8. Stain results are summarized in Table 2.

**Table 2:** Summary of stain results

Stain	Result
CD1a	Negative
CD2	Negative
CD3	Positive (cytoplasmic)
CD4	Positive
CD5	Negative
CD7	Negative
CD8	Negative
CD10	Negative
CD19	Negative
CD20	Negative
CD30	Negative
CD33	Negative
CD34	Negative
CD45	Negative
CD56	Negative
CD79a	Weak positive (variable, up to 10% multifocally)
CD117	Negative
CD138	Positive (strong, diffuse)
ALK1	Negative
BCL2	Positive
BCL6	Negative
CAM5.2	Negative
C-MYC	Positive
Cyclin D1	Negative
Granzyme B	Negative
Ki-67	95%
MUM1	Positive
Myeloperoxidase	Negative
Pancytokeratin	Negative
TdT	Negative
TIA1	Negative
Vimentin	Positive
Chromogenic in situ hybridization kappa	Negative
Chromogenic in situ hybridization lambda	Positive
Chromogenic in situ hybridization EBV	Negative

The morphologic and immunohistochemical findings, in the context of the patient’s medical history, represent extramedullary relapse in the GI tract of MM with aberrant expression of CD3.

The patient was treated with a regimen of carfilzomib 27mg on days 1, 8, and 15, pomalidomide 4mg, three weeks on and one week off, and dexamethasone 20mg weekly. He subsequently developed pancytopenia secondary to chemotherapy with complications from infections that delayed treatment. Ultimately, he developed lytic lesions on the distal right femur, hypercalcemia, and pneumonia. The decision to transition to hospice and palliative care was made.

## Discussion

Extramedullary relapse of MM in the GI tract is a rare but clinically significant phenomenon associated with a worse prognosis and more complex cytogenetics. Due to its rarity, most published articles are single case reports. A study of 24 cases of MM with involvement of the GI tract reported the liver as the most common location involved followed by the pancreas, stomach, peritoneum with malignant ascites, colon, rectum, duodenum, and ileum [5]. The addition of CD3 positivity in our case highlights a noteworthy presentation of relapsed MM in the GI tract, raising important considerations for differential diagnoses and diagnostic workup.

Extramedullary relapse of MM denotes a more aggressive clinical course with higher grade and plasmablastic or undifferentiated histology [6,7]. These histologic features were consistent with our case and yielded a broad differential diagnosis including poorly differentiated carcinoma and high-grade hematologic neoplasms. The typical panel of stains to narrow down a broad differential typically includes a combination of cytokeratins and general B-cell and T-cell markers such as CD20 and CD3, respectively; CD138, kappa, and lambda to assess for plasma cells are not usually included initially. In a situation where CD3 is positive with undifferentiated morphology, there is a risk of confusion with a peripheral T-cell lymphoma and, in fact, Pan et al. reported some cases to have been misclassified as such [8]. In our case, the lambda light chain restriction confirmed the B-cell lineage despite the expression of two T-cell antigens: CD3 and CD4. Other lymphomas with plasmablastic morphology that may aberrantly express T-cell markers include plasmablastic lymphoma and ALK-positive large B-cell lymphoma, which are typically EBV-positive and ALK-positive, respectively; however, our case was negative for both markers.

T-cell markers have been reported to be aberrantly expressed in both small B-cell lymphomas and diffuse large B-cell lymphomas (DLBCL), the most common marker being CD2 in both groups [9,10]. CD3 is often designated as a lineage-specific pan-T-cell marker but has been rarely reported in some cases of multiple myeloma with plasmablastic morphology and plasmablastic lymphoma. Other reported B-cell neoplasms with CD3 positivity include DLBCL, Burkitt lymphoma, and grade 3A follicular lymphoma [11].

The underlying mechanism for this aberrant T-cell marker expression on B-cell neoplasms is unclear. Several hypotheses have been proposed such as EBV infection, neoplasm transdifferentiation, downregulation of B-cell transcriptional factors, and the sensitivity and specificity of the CD3 antibody itself [8]. Further studies are needed to assist in elucidating the mechanism.

In our case, the patient’s relapsed MM was extremely unusual in that it expressed CD3, a rare occurrence, and presented with undifferentiated morphology while relapsing in an unexpected extramedullary location. Our knowledge of the patient’s prior MM diagnosis was advantageous; however, clinical history is not always readily available. In such cases, it is important to extend the typical immunohistochemical panel to include not only standard B-cell and T-cell markers but also plasma cell markers like CD138 and an assessment of kappa and lambda light chain clonality. This approach helps avoid misclassification as a peripheral T-cell lymphoma, which could impact therapeutic decisions and patient outcomes. This case also raises several unanswered questions about aberrant marker expression in multiple myeloma. For example, do these markers at initial diagnosis or at relapse have prognostic significance? Also, do these markers play a role in the pathogenesis of specific subsets of multiple myeloma? CD3 is a known signaling molecule that promotes T-cell proliferation. It is conceivable, then, that aberrant CD3 expression could promote the proliferation of neoplastic plasma cells as well. Future studies should be designed to help answer these questions.

## Conclusions

The diagnosis of MM from a histologic standpoint is usually straightforward if the classic histologic appearance is evident. However, in the absence of classic features, the diagnosis can be more challenging, and pitfalls are a risk. It is important for the pathologist to keep in mind that lineage infidelity may occur in poorly differentiated hematologic neoplasms and to broaden their immunohistochemical panel to include multiple B-cell and T-cell markers and consider including CD138, kappa, and lambda to rule out a plasma cell neoplasm.
